# Cognitive paths from trauma to posttraumatic stress disorder: a prospective study of Ehlers and Clark's model in survivors of assaults or road traffic collisions

**DOI:** 10.1017/S0033291719002253

**Published:** 2020-10

**Authors:** Esther T. Beierl, Inga Böllinghaus, David M. Clark, Edward Glucksman, Anke Ehlers

**Affiliations:** 1 University of Oxford, Oxford, UK; 2 King's College London, London, UK; 3 Oxford Health NHS Foundation Trust, Oxford, UK; 4 King's College Hospital NHS Foundation Trust, London, UK

**Keywords:** Appraisals, cognitive processing, dissociation, memory, posttraumatic stress disorder, rumination

## Abstract

**Background:**

Individual differences in cognitive responses to trauma may represent modifiable risk factors that could allow early identification, targeted early treatment and possibly prevention of chronic posttraumatic stress disorder (PTSD). Ehlers and Clark's cognitive model of PTSD suggests that negative appraisals, disjointed trauma memories, and unhelpful coping strategies maintain PTSD. These are thought to be influenced by cognitive processing during trauma. The aim of this study was to test this model prospectively with path analyses.

**Methods:**

Participants (*N* = 828) were recruited from an emergency department following injury in a violent assault or road traffic collision and 700 participated in the 6-month assessments. Cognitive processing was assessed shortly after the event, negative appraisals, disjointed memories, and unhelpful coping strategies at 1 month, persistent PTSD symptom severity at 6 months, and early PTSD symptom severity at 2 weeks.

**Results:**

Cognitive variables, with trauma type and gender, explained 52% of the variance in PTSD symptom severity at 6 months. Including early symptom severity in the model did not explain more variance (53%). Early PTSD symptom severity, with trauma type and gender, only predicted 40%. Negative appraisals and disjointed memories predicted persistent symptom severity both directly and indirectly via unhelpful strategies. Peritraumatic processing predicted persistent symptom severity mainly indirectly. The effects of trauma type and gender were fully mediated by the cognitive factors.

**Conclusions:**

The results are consistent with theoretically derived predictions and support cognitive factors as indicators of risk for chronic PTSD and as a target for the treatment and prevention of PTSD.

## Introduction

**Fig. 1.**
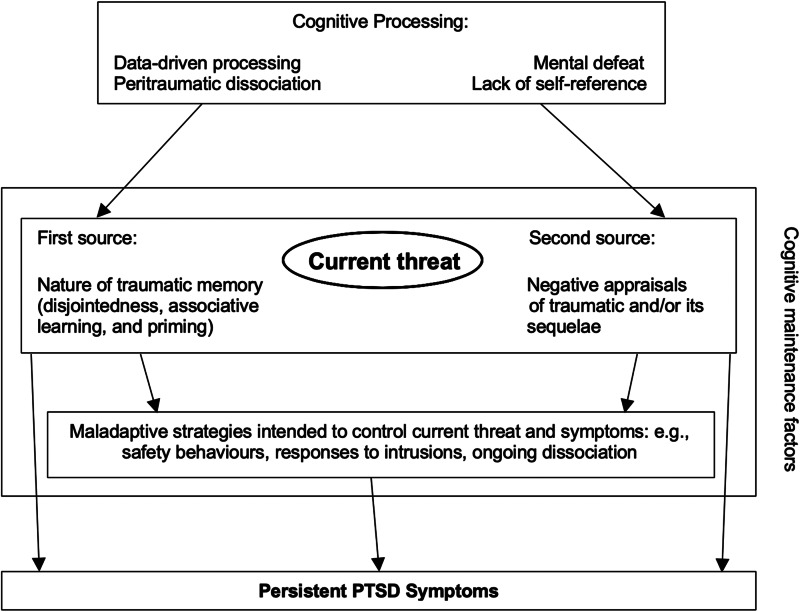
Conceptual figure of cognitive factors leading to chronic PTSD according to Ehlers and Clark's (2000) model.

Although most people are exposed to one or more traumatic events during their life time (Benjet *et al*., 2016; Kessler *et al*., 2017), only a minority develop PTSD, of whom nearly half recover without treatment during the following months (Morina *et al*., 2014). This raises the question of how to best identify the significant minority of individuals who develop chronic PTSD. Ehlers and Clark's (2000) model of PTSD (see Fig. 1) was developed to explain why some people do not recover after trauma and to identify *maintaining* factors that can be modified in treatment. The model suggests that chronic PTSD develops if trauma survivors process the traumatic event in a way that poses a serious *current* threat. The perceived threat has two sources: First, people with chronic PTSD show *excessively negative appraisals* (personal meanings) of the trauma and/or its sequelae. These appraisals are idiosyncratic and commonly include both appraisals about the self, such as ‘I am inadequate', and appraisals of other people/the world, e.g. ‘People cannot be trusted’. Second, the *trauma memory is disjointed* and poorly elaborated. Together with high perceptual priming and strong associative learning for stimuli present during the trauma, this leads to easy cue-driven triggering of memories (Ehlers *et al*., 2006, 2010; for a review see Marks *et al*., 2018). These memories are retrieved without a context and retain the original highly threatening meanings, as they are poorly linked with other information in memory that would put them into perspective (e.g. ‘I did not die’; Ehlers and Clark, 2000). Memory characteristics and negative appraisals are assumed to have a reciprocal relationship.

Ehlers and Clark's (2000) model further proposes that the sense of current threat motivates a series of *behavioural and cognitive strategies* that are intended to reduce the current threat but maintain the symptoms. These strategies include excessive precautions (safety behaviours), maladaptive responses to intrusive memories (thought suppression, rumination, intentional numbing), and persistent dissociation.

Thus, the core hypothesis of Ehlers and Clark's model is that appraisals, disjointed memories, and unhelpful strategies *maintain* PTSD. Several studies have found that these cognitive factors predict chronic PTSD (appraisals: e.g. Dunmore *et al*., 2001; Bryant and Guthrie, 2005; Ehring *et al*., 2008; Lancaster *et al*., 2011; Freeman *et al*., 2013; for a review see Brown *et al*., 2019; disjointed memories: e.g. Murray *et al*., 2002; Halligan *et al*., 2003; Freeman *et al*., 2013; safety behaviours: Dunmore *et al*., 2001; Ehring *et al*., 2008, responses to intrusions: Ehlers *et al*., 1998; Kleim *et al*., 2012*a*, Freeman *et al*., 2013; persistent dissociation: Murray *et al*., 2002; Halligan *et al*., 2003; Kleim *et al*., 2012*a*).

Ehlers and Clark's (2000) model further hypothesises that *cognitive processing* during the trauma predicts the *onset* of PTSD symptoms as they influence appraisals and memory characteristics. Four styles of problematic processing are specified: *Data-driven processing* refers to a preferential processing of perceptual information during the trauma (as opposed to its meaning) and is thought to increase the disjointedness of trauma memories and perceptual priming. *Lack of self-referent processing* (i.e. an inability of processing the experience as part of the continuum of autobiographical experiences) and *dissociation* (derealisation and depersonalisation) are also thought to contribute to the disjointedness of trauma memories. *Mental defeat* (i.e. a perceived loss of all autonomy during trauma), and lack of self-referent processing are thought to facilitate appraisals about a negative view of oneself and perceived permanent change. Several studies showed that these patterns of cognitive processing predict PTSD (e.g. data-driven processing: Halligan *et al*., 2003; Ehring *et al*., 2008; Freeman *et al*., 2013; dissociation: Ozer *et al*., 2003; lack of self-referent processing: Halligan *et al*., 2003; Freeman *et al*., 2013; Kleim *et al*., 2012*a*; mental defeat: Dunmore *et al*., 2001; Kleim *et al*., 2012*a*).

The aim of the current study was to test prospectively the direct and indirect paths by which the cognitive factors specified in Ehlers and Clark's (2000) model lead to persistent PTSD symptoms at 6 months after trauma. While earlier studies provided evidence that subsets of these cognitive variables predict chronic PTSD (e.g. Lancaster *et al*., 2011; Ehring *et al*., 2008; Kleim *et al*., 2012*a*) and also do so when controlling for early symptom severity (Ehlers *et al*., 1998; Kleim *et al*., 2007; Ehring *et al*., 2008), the present study used a more comprehensive set of measures and had a larger sample size that allowed testing the serial mediation effects proposed by Ehlers and Clark (2000). As a first step, we tested the central model of the maintenance of PTSD and hypothesized that negative appraisals and disjointed memories are correlated, and each predict persistent PTSD symptom severity directly and indirectly via unhelpful strategies (safety behaviours, responses to intrusions, and persistent dissociation). In the second step, we included the hypothesized role of cognitive processing styles during trauma in influencing appraisals and memory characteristics. We predicted that these are correlated, and each indirectly predicts PTSD symptom severity at 6 months, via different pathways: data-driven processing and dissociation via greater disjointedness of trauma memories, mental defeat via the severity of negative appraisals about the self, and lack of self-referent processing via both appraisals and disjointed memories. Two additional models aimed to test the clinically relevant question whether the maintaining cognitive factors together with early symptom severity after trauma (Step 4) explain more variance in persistent symptom severity at 6 months than early symptoms at 2 weeks alone (Step 3).

We included trauma type and gender in the path models as survivors of interpersonal traumas (Kessler *et al*., 2017) and women (Olff *et al*., 2007) are at a higher risk of experiencing PTSD symptoms and would therefore be expected to also score higher on the cognitive factors.

## Methods

### Sample

Participants were recruited from the Emergency Department of King's College Hospital London following injury in a violent assault or road traffic collision (RTC). They were invited to participate in the study either by a research assistant when attending the Emergency Department or by letter, followed by a phone call. Inclusion criteria were: Aged between 18 and 65, trauma meets DSM-IV criteria for PTSD (American Psychiatric Association, 2000), able to read and write in English. Exclusion criteria were current psychosis or substance dependence, unable to remember the trauma, or assault in the context of ongoing domestic violence. These criteria were asked by structured questions at recruitment. Of 1291 people who were interested and suitable for the study, 828 provided data, and 700 participants provided PTSD symptom data 6 months after the traumatic event (see missing data analyses in the online Supplementary Material). Two smaller subsamples of the participants in this study also participated in studies of heart rate responses to pictures (Ehlers *et al*., 2010; Sündermann *et al*., 2010). There is no overlap in participants with other earlier studies conducted at the same emergency department (Kleim *et al*., 2007, 2012*a*; Ehring *et al*., 2008; Freeman *et al*., 2013). Participants' demographic characteristics are shown in Table 1. All participants experienced a violent physical assault, and for 3 the assault also had a sexual element.

**Table 1.** Demographic characteristics and trauma types of the participants (*N* = 700)

Variable	*n* (*%*)	*M* (s.d.)
Age (years)		35.08 (11.04)
Trauma type		
Assault	308 (44)	
Road traffic collision	392 (56)	
Gender		
Male	439 (63)	
Female	261 (37)	
Trauma type x gender		
Assault and female	89 (13)	
Assault and male	219 (31)	
Road traffic collision and female	172 (25)	
Road traffic collision and male	220 (31)	
Ethnic group		
Caucasian/white	363 (52)	
Black	210 (30)	
(Indo)Pacific Asian	21 (3)	
Other	72 (10)	
Missing values	34 (5)	
Marital Status		
Single	406 (58)	
Married	179 (25)	
Divorced	62 (9)	
Widowed	6 (1)	
Missing values	47 (7)	
Level of education		
University degree	184 (26)	
Vocational degree	22 (3)	
A-level	123 (18)	
GCSE	186 (27)	
No exams	66 (9)	
Other	63 (9)	
Missing values	56 (8)	
Employment status		
Employed	385 (55)	
Self-employed	70 (10)	
Student	61 (9)	
Unemployed	76 (11)	
Homemaker	11 (2)	
Disability benefits	19 (3)	
On sick leave	29 (4)	
Retired	5 (1)	
Other	8 (1)	
Missing values	36 (5)	

### Measurements

#### Cognitive processing

Peritraumatic cognitive processing was assessed using a short version of the Cognitive Processing Questionnaire (Dunmore *et al*., 2001; Halligan *et al*., 2003). The short questionnaire contains a total of 22 items (*α* = 0.96) measuring data-driven processing (6 items, *α* = 0.91), lack of self-referent processing (5 items, *α* = 0.91), dissociation (i.e. derealisation and depersonalisation, 5 items, *α* = 0.88), and mental defeat during the traumatic event (6 items, *α* = 0.94).

#### Appraisals of the traumatic event and its sequelae

A 34-item version of the Post-traumatic Cognitions Inventory (Foa *et al*., 1999) assessed negative appraisals of the trauma and its aftermath, *α* = 0.95.

#### Trauma memories

The disjointed quality of participants' trauma memories was assessed with a short version (Sachschal *et al*., 2019) of the Trauma Memory Questionnaire (Halligan *et al*., 2003), which included 4 items, *α* = 0.80.

#### Safety behaviours

Excessive precautions were assessed with a 13-item version of the Safety Behaviour Questionnaire (Dunmore *et al*., 1999; 2001), *α* = 0.91.

#### Responses to intrusive memories

The Responses to Intrusions Questionnaire (Clohessy and Ehlers, 1999; Murray *et al*., 2002) contains 19 items (*α* = 0.91) assessing thought suppression (6 items), rumination (8 items), and emotional numbing (5 items).

#### Persistent dissociation

The State Dissociation Questionnaire (Murray *et al*., 2002) comprises 9 items, *α* = 0.94.

#### PTSD symptom severity

The Posttraumatic Diagnostic Scale (PDS; Foa *et al*., 1997) assessed the severity of the 17 PTSD symptoms specified in DSM-IV-TR (American Psychiatric Association, 2000) at 2 weeks (*α* = 0.93). PTSD symptom severity at 6 months was assessed using the Posttraumatic Diagnostic Scale, *α* = 0.95, and the PTSD Symptom Scale-Interview (PSSI; Foa *et al*., 1993; average ICC = 0.98); a composite score of both z-standardised measures was computed.

#### Further predictors

A trained specialist nurse coded injury severity from hospital records with the Injury Severity Score (Baker *et al*., 1974). Trauma type was coded as 1 (*assault*) and 0 (*RTC*), gender as 1 (*female*) and 0 (*male*).

### Procedure

The study was approved by the Institute of Psychiatry and South London and Maudsley Joint Ethics Committee (150/04) and the King's College Hospital Ethics Committee in London, UK (020/03). Participants gave written informed consent. Participants completed the Cognitive Processing Questionnaire at recruitment either in the Emergency Department within hours after their treatment for injuries or by post (which took up to 10 days after the traumatic incident). Appraisals, memory, safety behaviours, responses to intrusions, and persistent dissociation were assessed at 1 month via questionnaires. PTSD symptom severity was assessed at 2 weeks via questionnaire (PDS) and at 6 months by both structured interview with a trained psychologist (PSSI) and the PDS. Example items for all measures are provided in online Supplementary Table S1. Participants were reimbursed £60 for their time.

### Statistical analyses

Path models (Wright, 1934) were specified, in which all hypothesised effects and correlations were freely estimated. Unstandardized (*b*) and standardized (*β*) estimates can be interpreted as in multiple regression. We evaluated model fit using the χ^2^-test statistic (Jöreskog, 1969; Bollen, 1989) and the fit indices CFI (Bentler, 1990), RMSEA (Steiger and Lind, 1980), and SRMR (Jöreskog and Sörbom, 1981). As the variables had 4–7 response categories, they were treated as continuous for the use of a robust Maximum Likelihood methodology (see Rhemtulla *et al*., 2012). For the computation of prorated scale mean scores, at least half of the items had to be answered by the participant, otherwise the mean scores were regarded as missing data. Observed values deviated from univariate normality (see Table 2 for descriptive statistics) and were not multivariate normally distributed (Mardia's test *p* < 0.001). Therefore, we corrected the χ^2^-test statistic (method by Yuan and Bentler, 1998, 2000) and nonparametrically bootstrapped (Efron and Tibshirani, 1986) standard errors, which is also one method to determine significance of the indirect effects (Bollen and Stine, 1990). 95% adjusted bootstrap percentile confidence intervals for the indirect effects are reported (MacKinnon *et al*., 2004). We used the Full Information Maximum Likelihood algorithm (Arbuckle, 1996), so that all participants with 6-month data could be included in the analysis (Enders and Bandalos, 2001). The assumption of normality of the residuals of the models was found to be satisfactory. Results of a Monte Carlo power simulation (Muthén and Muthén, 2009) indicated sufficient power to detect the hypothesised effects.

**Table 2.** Descriptive statistics of the variables of the cognitive model and additional measures (*N* = 700)

Variables	*n*	*M*	s.d.	Skewness (s.e.)	Kurtosis (s.e.)
Cognitive model					
Mental defeat	678	0.79	1.13	1.46 (0.09)	0.95 (0.19)
Lack of self-reference	679	1.12	1.22	0.95 (0.09)	−0.31 (0.19)
Peritraumatic dissociation	678	1.61	1.23	0.37 (0.09)	−1.05 (0.19)
Data-driven processing	679	1.98	1.17	0.05 (0.09)	−1.13 (0.19)
Appraisals of the traumatic event/ its sequelae	598	2.30	1.10	1.33 (0.10)	1.50 (0.20)
Memory characteristics	603	1.07	0.97	0.81 (0.10)	−0.16 (0.20)
Safety behaviours	588	1.60	0.67	−0.13 (0.10)	−0.69 (0.20)
Responses to intrusions	608	1.07	0.62	0.27 (0.10)	−0.58 (0.20)
Persistent dissociation	597	0.65	0.85	1.63 (0.10)	2.03 (0.20)
PTSD symptoms at 6 months (composite self-report, clinician rating)^a^	700	15.00	10.22	0.90 (0.09)	−0.11 (0.19)
Additional measures					
PTSD symptoms at 2 weeks (self-report)^a^	630	19.84	12.61	0.41 (0.09)	−0.75 (0.19)
Injury severity	573	2.04	2.36	3.39 (0.10)	15.47 (0.20)

*Note*. Mental defeat, lack of self-reference, peritraumatic dissociation, data-driven processing: 5-point Likert scale; Appraisals of the traumatic event/ its sequelae: 7-point Likert scale; memory characteristics: 5-point Likert scale; safety behaviours: 4-point Likert scale; responses to intrusions: 4-point Likert scale; persistent dissociation: 5-point Likert scale.

a For PTSD symptoms, the sum score is reported (4-point Likert scale); for all other measures, the mean of the scale is reported. Higher scores on all measures represent higher endorsements.

Data were analysed using *RStudio* (RStudioTeam, 2018) and the packages *lavaan* (Rosseel, 2012), *sjstats* (Lüdecke, 2018), *skimr* (McNamara *et al*., 2019), *MVN* (Korkmaz *et al*., 2016), and *irr* (Gamer *et al*., 2019). The R code can be retrieved from osf.io/k258j/.

## Results

### Preliminary analyses

Recruitment method neither influenced persistent PTSD symptom severity 6 months post-trauma, *b* = −0.05, *β* = −0.02, *p* = 0.41, nor did it moderate the prediction of persistent symptom severity by peritraumatic processing, *b* = 0.01, *β* = 0.003, *p* = 0.92. Assault survivors experienced more severe PTSD symptoms at 6 months than RTC survivors, *F*_(1,696)_ = 38.10, *p* < 0.001, *η*_*p*_^2^ = 0.051. Women experienced more severe persistent symptoms than men, *F*_(1,696)_ = 5.26, *p* = 0.020, *η*_*p*_^2^ = 0.007. Trauma type and gender interacted with each other, *F*_(1,696)_ = 9.23, *p* = 0.002, *η*_*p*_^2^ = 0.013. The difference in persistent PTSD symptom severity between men and women was greater for assault than RTC survivors, *p*_*adj*_ = 0.005 (see Table 1). Severity of injury did not predict symptom severity at 6 months, *b* = 0.01, *β* = 0.02, *p* = 0.55. 14 participants reported that they had received an evidence-based treatment due to the trauma, but this did not predict symptom severity at 6 months over and above early symptom severity, *F*_(1,517)_ = 326.92, *p* < 0.001, *R*^2^ = 0.39; Δ*F*_(1,516)_ = 0.33, *p* = 0.57, Δ*R*^2^ = 0.00.

### Path analyses

#### Model fit of path analytic test of Ehlers and Clark's model of PTSD

The first step tested the core maintenance model (Ehlers and Clark, 2000) which states that appraisals, disjointed memories and unhelpful strategies predict persistent PTSD symptom severity at 6 months post-trauma (see online Supplementary Fig. S1). Non-significant effects of trauma type and gender were set to zero. Modification indices suggested a correlation between safety behaviours and responses to intrusions, which was included. The resulting model fit the data, χ^2^(11) = 14.74, χ^2^/df = 1.34, *p* = 0.20, CFI = 1.00, RMSEA = 0.02 (0.00–0.05), SRMR = 0.02, and explained 52% of variance in PTSD symptom severity at 6 months.

**Fig. 2.**
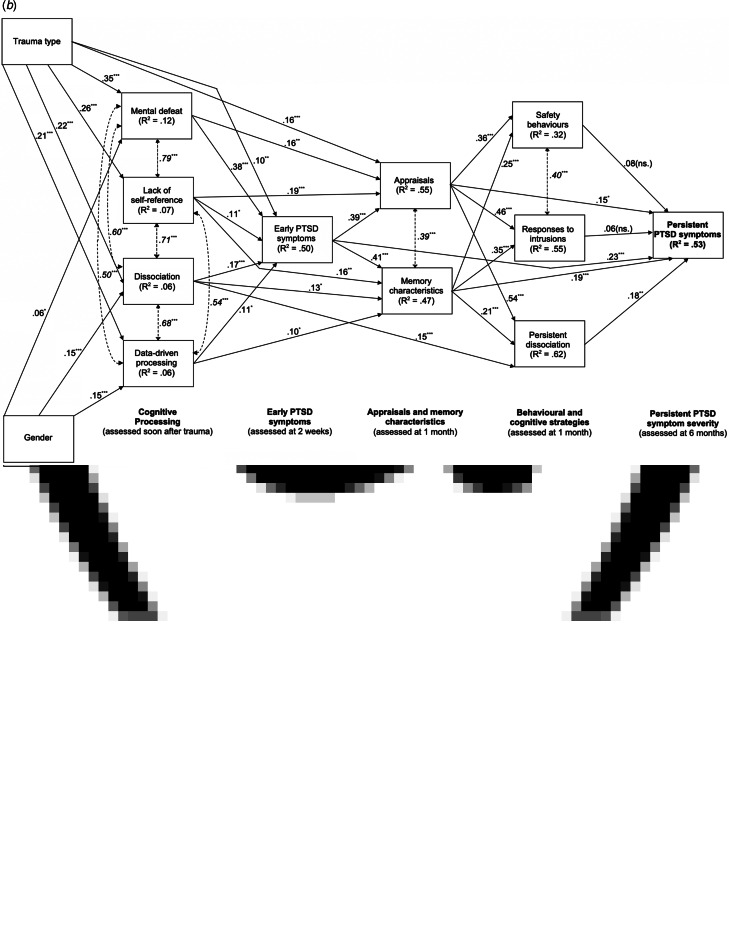
(*a*) Path model for Ehlers and Clark's (2000) cognitive model of PTSD. Cognitive processing during trauma was assessed within hours up to 10 days after the traumatic event; appraisals, memory characteristics, safety behaviours, responses to intrusions and ongoing dissociation were assessed 1 month after the traumatic event, and persistent posttraumatic stress disorder symptoms were assessed 6 months after the traumatic event. Trauma type is coded as 0 (road traffic collision) and 1 (assault), gender is coded as 0 (male) and 1 (female). Continuous lines with arrows pointing in one direction show standardized path coefficients, dashed lines with double headed arrows show correlations (in italics). *R*^2^ = percentage of explained variance, ***/**/* = *p* < 0.001/<0.01/<0.05. (*b*) Path diagram of the cognitive model extended by early symptoms. Thick continuous lines with arrows pointing in one direction show standardized path coefficients for the predictions of the cognitive factors and early PTSD symptom severity, thin continuous lines with arrows pointing in one direction show standardized path coefficients for effects of trauma type (coded as 0 = road traffic collision and 1 = assault) and gender (coded as 0 = male and 1 = female), and dashed lines with double headed arrows and values in italics font show correlations. *R*^2^ = percentage of explained variance, ***/**/* = *p* < 0.001/<0.01/<0.05.

The second step included the hypothesised effects of the four cognitive processing styles in the model. The non-significant effect of gender on lack of self-referent processing was set to zero. After requesting the modification indices, paths from peritraumatic dissociation to chronic dissociation, from mental defeat to disjointed memories, and from data-driven processing to PTSD symptoms at 6 months were included in the model. The resulting path model fit the data, χ^2^(30) = 37.96, χ^2^/df = 1.27, *p* = 0.15, CFI = 1.00, RMSEA = 0.02 (0.00–0.04), SRMR = 0.03 (see Fig. 2*a*), and explained 52% of variance in PTSD symptom severity at 6 months.

#### Model parameters

All hypothesised path coefficients were significant, providing empirical support for Ehlers and Clark's (2000) model (see Fig. 2*a*). Table 3 summarises the direct, indirect, and total effects.

**Table 3.** Indirect, direct and total effects of variables derived from the cognitive model (*N* = 700)

Effects	*b* (95% CI)	*β*
*Peritraumatic processing*		
Mental defeat		
Indirect effect of mental defeat	0.145 (0.080–0.212)	0.169
Total effect of mental defeat	0.146 (0.080–0.212)	0.169
Lack of self-reference		
Indirect effect of lack of self-reference	0.163 (0.106–0.224)	0.206
Total effect of lack of self-reference	0.163 (0.106–0.224)	0.206
Peritraumatic dissociation		
Indirect effect of peritraumatic dissociation	0.065 (0.031–0.099)	0.082
Total effect of peritraumatic dissociation	0.065 (0.031–0.099)	0.082
Data-driven processing		
Indirect effect of data-driven processing	0.033 (0.009–0.063)	0.040
Direct effect of data-driven processing (see Fig. 2a)	0.063 (0.015–0.117)	0.076
Total effect of data-driven processing	0.096 (0.042–0.156)	0.116
Sum of the effects of the four peritraumatic processing styles	0.470 (0.406–0.526)	0.574
Appraisals		
Indirect effect of appraisals	0.161 (0.089–0.236)	0.187
Direct effect of appraisals (see Fig. 2a)	0.175 (0.067–0.283)	0.204
Total effect of appraisals	0.336 (0.262–0.413)	0.391
Memory disjointedness		
Indirect effect of memory disjointedness	0.099 (0.058–0.147)	0.100
Direct effect of memory disjointedness (see Fig. 2*a*)	0.212 (0.102–0.315)	0.215
Total effect of memory disjointedness	0.311 (0.209–0.406)	0.314
Maintaining strategies		
Direct effect of safety behaviours (see Fig. 2*a*)	0.122 (0.018–0.234)	0.085
Direct effect of responses to intrusions (see Fig. 2*a*)	0.161 (0.025–0.308)	0.105
Direct effect of persistent dissociation (see Fig. 2*a*)	0.221 (0.089–0.354)	0.198
Further predictors		
Indirect effect of trauma type	0.436 (0.343–0.527)	0.224
Total effect of trauma type	0.436 (0.343–0.527)	0.224
Indirect effect of gender	0.077 (0.046–0.116)	0.038
Total effect of gender	0.077 (0.046–0.116)	0.038

*Note.* Indirect effects are calculated by multiplying the paths that lead from the variable to persistent PTSD symptoms at 6 months via specific mediators. Adjusted bootstrap percentile confidence intervals are reported. Confidence intervals not including zero indicate significant parameter estimates.

*Direct and indirect effects of peritraumatic processing*. Mental defeat, *β* = 0.17, *p* < 0.001, and lack of self-referent processing, *β* = 0.21, *p* < 0.001, indirectly predicted PTSD symptom severity at 6 months via both disjointed memories and negative appraisals, and via the maintaining strategies (see Fig. 2*a* and Table 3). Peritraumatic dissociation, *β* = 0.08, *p* < 0.001, and data-driven processing, *β* = 0.04, *p* = 0.016, indirectly predicted persistent symptom severity via disjointed memories and the three maintaining strategies (see Fig. 2*a* and Table 3). In addition to the hypothesized effects, there was a direct path from data-driven processing to persistent symptom severity, *β* = 0.08, *p* = 0.020, and a significant path from mental defeat to disjointed memories, *β* = 0.13, *p* = 0.019 (see Table 3).

*Direct and indirect effects of appraisals*. Negative appraisals at 1 month were predicted by the severity of mental defeat and lack of self-referent processing (see Fig. 2*a*). Degree of endorsement of negative appraisals at 1 month directly predicted PTSD severity at 6 months, *β* = 0.20, *p* = 0.001 (see Fig. 2*a* and Table 3). There were indirect effects via each of the three maintaining strategies: The total indirect effect of negative appraisals on PTSD symptoms at 6 months via safety behaviours, responses to intrusions, and persistent dissociation was *β* = 0.19, *p* < 0.001, and the total effect *β* = 0.39, *p* < 0.001 (see Table 3).

*Direct and indirect effects of disjointed memories*. Disjointed memories at 1 month were predicted by all four cognitive processing styles (see Fig. 2*a*). Severity of memory disjointedness at 1 month directly predicted PTSD severity at 6 months, *β* = 0.21, *p* < 0.001 (see Table 3). There were indirect effects via each of the three types of maintaining strategies: The total indirect effect of memory disjointedness (see Table 3) via safety behaviours, responses to intrusions, and persistent dissociation was *β* = 0.10, *p* < 0.001, and the total effect *β* = 0.31, *p* < 0.001 (see Table 3).

*Effects of maintaining strategies*. Each of the maintaining strategies at 1 month, safety behaviours, *β* = 0.09, *p* = 0.018, responses to intrusions, *β* = 0.11, *p* = 0.026, and persistent dissociation, *β* = 0.20, *p* = 0.001, predicted the severity of PTSD symptoms at 6 months (see Fig. 2*a* and Table 3).

*Correlations*. Mental defeat, lack of self-reference, peritraumatic dissociation, and data-driven processing correlated highly, and participants who endorsed negative appraisals to a greater extent also reported more disjointed trauma memories (see Fig. 2*a*). Safety behaviours were positively associated with responses to intrusions (see Fig. 2*a*).

*Effects of type of trauma and gender*. Having experienced an assault compared to an RTC was associated with more severe mental defeat, *b* = 0.78, *β* = 0.35, *p* < 0.001, lack of self-referent processing, *b* = 0.63, *β* = 0.26, *p* < 0.001, peritraumatic dissociation, *b* = 0.54, *β* = 0.22, *p* < 0.001, data-driven processing, *b* = 0.50, *β* = 0.21, *p* < 0.001, and more negative appraisals, *b* = 0.40, *β* = 0.18, *p* < 0.001 (see Fig. 2*a*). Women scored higher on mental defeat, *b* = 0.13, *β* = 0.06, *p* = 0.009, peritraumatic dissociation, *b* = 0.37, *β* = 0.15, *p* < 0.001, and data-driven processing, *b* = 0.35, *β* = 0.15, *p* < 0.001, than men (see Fig. 2*a*). Direct effects of trauma type or gender on PTSD symptoms at 6 months were non-significant, i.e. their effects on PTSD symptom severity were fully mediated by cognitive factors (see Fig. 2*a* and Table 3).

#### Role of early symptom severity in the prediction of persistent PTSD symptom severity

We fit a third and fourth path model to the data to investigate the relative role of early symptom severity and cognitive factors in the prediction of persistent symptom severity. The third model investigated the prediction of persistent PTSD symptom severity at 6 months by early symptom severity at 2 weeks, trauma type, and gender (see online Supplementary Fig. S2). The model fit the data, χ^2^(2) = 1.50, χ^2^/df = 0.75, *p* = 0.47, CFI = 1.00, RMSEA = 0.00 (0.00–0.07), SRMR = 0.01, and the three predictors explained 40% of variance in persistent PTSD symptom severity.

The fourth model included early PTSD symptoms in the path analysis of the cognitive model from Step 2 (see Fig. 2*b*). The non-significant direct path of data-driven processing and the non-significant effect of mental defeat on memory disjointedness were set to zero. The resulting model explained 53% of variance in persistent PTSD symptom severity, χ^2^(36) = 91.99, χ^2^/df = 2.56, *p* < 0.001, CFI = 0.99, RMSEA = 0.05 (0.04–0.06), SRMR = 0.03.

Early symptom severity and the cognitive factors explained more variance than early symptom severity alone (53% compared to 40%, *z* = 4.72, *p* < 0.001, Step 4 compared to Step 3). However, early symptom severity and the cognitive factors did not explain more variance in persistent symptom severity than the cognitive factors alone (53% compared to 52%, *z* = 0.37, *p* = 0.36, Step 4 compared to Step 2).

## Discussion

This study was the first to comprehensively test the cognitive pathways to chronic PTSD symptoms specified in Ehlers and Clark's (2000) model of chronic PTSD in a prospective study of trauma survivors recruited in the first days after trauma. As predicted by the core model of the maintenance of PTSD symptoms (Step 1), negative appraisals of the traumatic event and/or its sequelae and disjointed memories at 1 month predicted symptom severity 6 months after the trauma directly and indirectly via maladaptive behavioural and cognitive coping strategies (of which safety behaviours, responses to intrusions, and persistent dissociation were tested here). Also in line with theoretical predictions, Step 2 showed that the degree of peritraumatic processing indirectly predicted PTSD symptom severity at 6 months via its effects on both, posttraumatic negative appraisals about the self and the world and the disjointedness of participants' trauma memories. In addition, data-driven processing during trauma also showed a small direct effect on PTSD symptoms at 6 months. Both path analytic models (Steps 1 and 2) explained 52% of the variance in PTSD symptom severity at 6 months after the trauma, which was a larger percentage than the percentage explained by early PTSD symptoms (40%, Step 3). Furthermore, the inclusion of early PTSD symptoms at 2 weeks post-trauma did not explain more variance in PTSD symptom severity at 6 months than the cognitive factors alone (53% for Step 4 *v.* 52% for Step 2, ns.). Thus, cognitive responses during trauma and in the first month afterwards strongly predict who will develop chronic PTSD, and have greater predictive power than symptom severity alone, which extends findings from previous prospective studies (e.g. Ehlers *et al*., 1998; Dunmore *et al*., 2001; Halligan *et al*., 2003; Kleim *et al*., 2007, 2012*a*; Ehring *et al*., 2008; Freeman *et al*., 2013).

These results have practical implications for both the decision to whom treatment should be offered and treatment targets. First, NICE treatment guidelines (2018) currently recommend a period of active monitoring for trauma survivors with subthreshold symptoms. Thus, the severity of symptoms is used to determine prognosis. This study suggests that assessing appraisals, memory characteristics and unhelpful coping strategies could improve the accuracy of prognostic judgements. Second, the result that the effects of peritraumatic processing on chronic PTSD symptom severity was nearly fully mediated by the maintaining factors (appraisals, memory characteristics, and unhelpful coping strategies) supports the importance to distinguish between onset and maintenance factors of PTSD as suggested by Ehlers and Clark (2000), and the emphasis on maintenance factors in treatment. The maintaining factors investigated here are modifiable and can be targeted with psychological treatments. Cognitive therapy for PTSD, which directly builds on Ehlers and Clark's model, is one of the evidence-based treatments for PTSD (e.g. Ehlers *et al*., 2003, 2005, 2014). Furthermore, it has been shown that changes in appraisals drive symptom change in this treatment (Kleim *et al*., 2013) as well as in prolonged exposure (Zalta *et al*., 2014). Thus, changing overly negative appraisals about the meaning of the trauma for the individual's view of themselves and the world appears to be an important treatment goal that is addressed in evidence-based psychological treatments of PTSD (see also Schnyder *et al*., 2015). Third, targeting the modifiable factors may offer the possibility for developing training programmes for the prevention of PTSD.

The results also are of theoretical interest. The inclusion of a measure of memory disjointedness demonstrated for the first time that although memory characteristics and appraisals were correlated, there are significant cognitive pathways to chronic PTSD that were mediated by memory disjointedness alone. The standardized total effect of memory disjointedness on chronic PTSD symptoms was *β* = 0.31. This is an important finding given that the nature of trauma memories has been the subject of considerable debate and supports theoretical models highlighting the role of perceptual processing and memories for trauma in PTSD (e.g. Ehlers and Clark, 2000; Brewin, 2014).

Negative appraisals of the self and the world at one month were important in the prediction of PTSD symptom severity at 6 months, with a total effect of *β* = 0.39. These findings are in line with other prospective studies (e.g. for a review see Brown *et al*., 2019) and several theoretical models of PTSD that emphasize the role of appraisals in the development and maintenance of PTSD (e.g. Foa *et al*., 1993; Resick and Schnicke, 1993; Ehlers and Clark, 2000).

The results also showed that the well-established effects of trauma type (Kessler *et al*., 2017) and gender (Olff *et al*., 2007) on PTSD symptoms were fully mediated by cognitive factors. In line with previous findings that women experience higher levels of dissociation and greater loss of control than men during trauma (Olff *et al*., 2007), women reported more pronounced peritraumatic processing, which put them at greater risk of PTSD than men. Having experienced an assault was associated with greater peritraumatic processing and endorsement of negative appraisals compared to RTCs. Interpersonal trauma is more likely to affect negative processing about the self (e.g. mental defeat and self-blame), which is linked to higher rates of PTSD than accidental trauma.

The path analysis further allowed us to test how the four styles of cognitive processing specified in Ehlers and Clark's (2000) model contribute to chronic PTSD symptom severity. In line with predictions from this model, the effect of dissociation and data-driven processing on chronic PTSD symptoms was mediated by the disjointed nature of the trauma memories, whereas the effect of mental defeat was mediated by its effect on appraisals, and the effect of lack of self-referent processing via both disjointed memories and negative appraisals.

The study has several strengths but also limitations. It included a more comprehensive set of cognitive measures than previous tests of Ehlers and Clark's model and could therefore analyse nearly all causal paths specified in the model, with the exception of two further memory processes specified in the theoretical model, strong and generalised associative learning and perceptual priming, which were not assessed. The direct effect of data-driven processing on chronic PTSD symptoms observed in Step 2 is likely to be mediated by enhanced perceptual priming, which has been demonstrated in previous studies (e.g. Ehlers *et al*., 2006). These are thought to explain the easy triggering of reexperiencing symptoms by a very wide range of stimuli in PTSD and have been shown to predict intrusive memories and chronic PTSD (e.g. Ehlers *et al*., 2010; Sündermann *et al*., 2010, 2013; Kleim *et al*., 2012*b*).

The decision to include trauma survivors with different trauma types increases the generalisability of the findings but also restricts the percentage of variance explained in chronic PTSD symptoms, which is likely to represent a lower bound estimate of the predictive power of the cognitive constructs (see Kleim *et al*., 2012*a*). Ehlers and Clark's (2000) model states that appraisals and unhelpful coping strategies used by an individual with PTSD are idiosyncratic and may vary by trauma type. The questionnaires used in this study could only assess common patterns, and thus represent incomplete assessments of an individual's appraisals and strategies. For example, we used a generic scale of safety behaviours and did not include a specific scale for safety behaviour around travel that is more predictive of PTSD in RTC survivors (Ehring *et al*., 2008). Even with the large sample size, we were not able to analyse separate path models for survivors by trauma type or gender, nor could we include these risk factors as moderators in the model. Finally, we also did not include early measures of negative appraisals and memory disjointedness, which would likely explain additional variance in early PTSD symptom severity.

In conclusion, the study supported the predictions from Ehlers and Clark's (2000) model of PTSD and suggests a focus on excessively negative appraisals, trauma memory characteristics and unhelpful coping strategies such as safety behaviours, rumination, thought suppression, emotional numbing, and ongoing dissociation in the early identification, treatment, and prevention of chronic PTSD.

## Supplementary material

For supplementary material accompanying this paper visit https://doi.org/10.1017/S0033291719002253. click here to view supplementary material
