# Supplementary material for: Cognitive paths from trauma to posttraumatic stress disorder: a prospective study of Ehlers and Clark's model in survivors of assaults or road traffic collisions
Source: Psychol Med. 2019 Sep 11;50(13):2172–81. doi: 10.1017/S0033291719002253 (PMC7557160; doi:10.1017/S0033291719002253)
Supplement: Supplementary file 1 [file S0033291719002253sup001.docx]

Supplemental Material

Cognitive paths from trauma to posttraumatic stress disorder: A prospective study of Ehlers and Clark’s model

in survivors of assaults or road traffic collisions

Esther T. Beierl, Inga Böllinghaus, David M. Clark, Edward Glucksman and Anke Ehlers

*Missing data analyses*

The sample of people who were suitable and interested in the study (*N* = 1291) consisted of 642 (50%) assault and 649 (50%) RTC survivors and 823 (64%) men and 467 (36%) women. The final sample of participants who provided PTSD symptom data after 6 months (*n* = 700) consisted of 308 (44%) assault and 392 (56%) RTC survivors and 439 (63%) men and 261 (37%) women (see Table 1 in the manuscript). Attrition from the sample of suitable and interested people to the final sample of participants who provided PTSD symptom data after 6 months versus those 592 cases who did not depended on trauma type (more assault survivors dropped out), χ^2^(1) =20.12, *p* < .001, but not on gender, χ^2^(1) = 0.72, *p* = .40.

Within the final sample of participants who provided PTSD symptom data at 6 months, 630 (90%) provided early PTSD symptom data at 2 weeks and 70 (10%) did not. Participants who did not provide early PTSD symptom data had higher PTSD symptoms at 6 months, *t*(81.62) = 3.39, *p* = .001.

**Table S1.** Example items for all questionnaires and subscales used separately in the study

| Questionnaire *(answer format)*  Subscales | Example items |
| --- | --- |
| **Cognitive Processing Questionnaire** *(5-point Likert scale)*  Mental defeat  Lack of self-reference    Peritraumatic dissociation    Data-driven processing  **Post-traumatic Cognitions Inventory** *(7-point Likert scale)*  **Trauma Memory Questionnaire** *(5-point*  *Likert scale)*  **Safety Behaviour Questionnaire** *(4-point*  *Likert Scale)*  **Responses to Intrusions Questionnaire** *(4-*  *point Likert Scale)*  **State Dissociation Questionnaire** *(5-point*  *Likert Scale)*  **Posttraumatic Diagnostic Scale/ PTSD Symptom Scale Interview** *(4-point*  *Likert Scale)* | ‘I felt no longer like a human being’  ‘I felt like I was a different person from the one I used to be’  ‘The world around me seemed strange or unreal’  ‘It was just like a stream of unconnected impressions following each other’  ‘My reactions since the event mean that I am losing my mind’  ‘To what extent did the memories seem to be isolated fragments that were disconnected from what happened immediately and afterwards?’  ‘I take precautions whatever I do’  ‘I dwell on how the accident/assault could have been prevented’  ‘I felt as if I was separate to my body and was watching it from outside’  ‘Reliving the accident/assault, acting or feeling as if it were happening again’ |

**
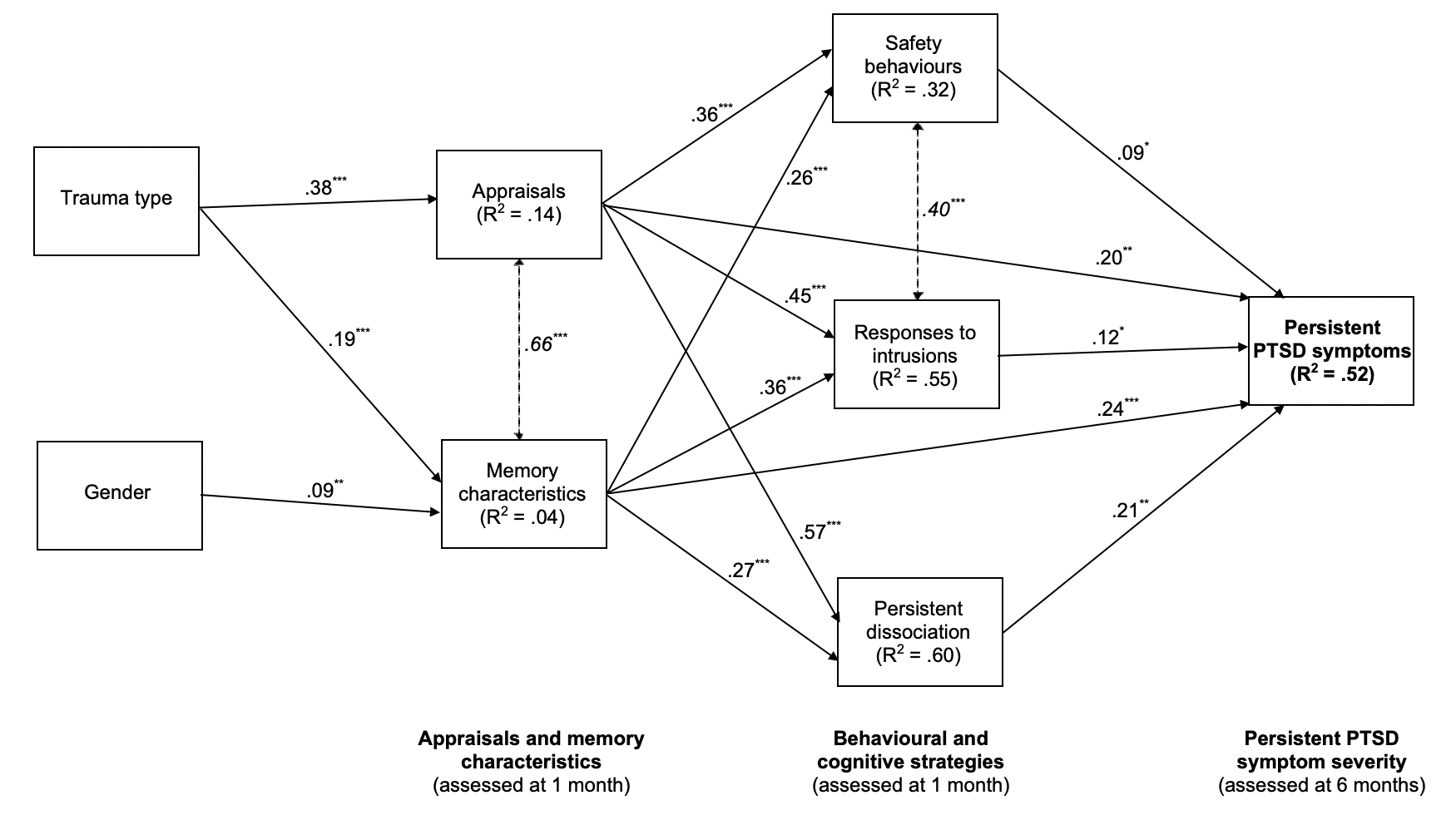
**

**Fig. S1.** Path model for maintenance of PTSD symptoms according to Ehlers and Clark (2000). Thick continuous lines with arrows pointing in one direction show standardized path coefficients for the main hypotheses regarding maintenance of PTSD symptoms according to Ehlers and Clark model, thin continuous lines with arrows pointing in one direction show standardized path coefficients for effects of trauma type and gender, and dashed lines with double headed arrows and values in italics font show correlations. Trauma type is coded as 0 (road traffic collision) and 1(assault), gender is coded as 0 (male) and 1 (female). R^2^ = percentage of explained variance, ***/**/* = *p* < .001/< .01/< .05.


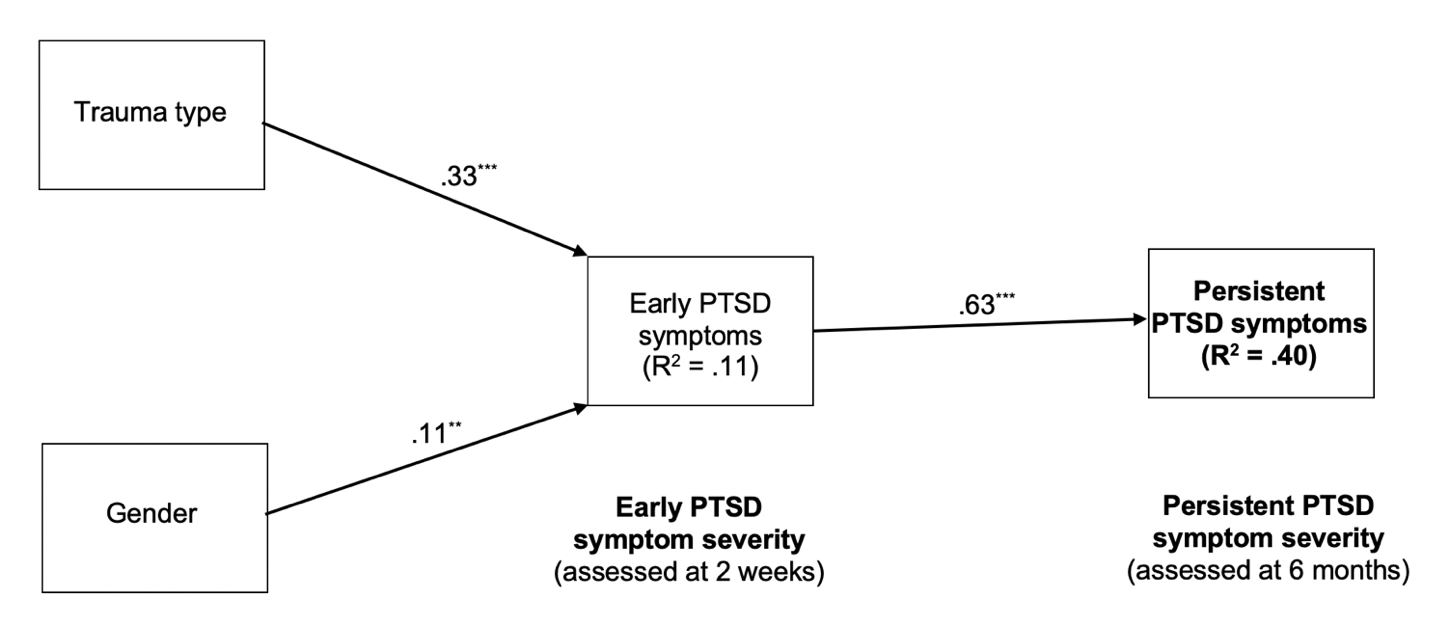


**Fig. S2.** Path model for early PTSD symptoms predicting persistent PTSD symptoms. Direct effects of trauma type (coded as 0 = road traffic collision and 1= assault) and gender (coded as 0 = male and 1 = female) were non-significant. Lines show standardized path coefficients. R^2^ = percentage of explained variance, ***/**/* = *p* < .001/< .01/< .05.
